# First detection of a *Mycobacterium tuberculosis* XDR clinical isolate harbouring an RpoB I491F mutation in a Ukrainian patient treated in Germany, October 2023

**DOI:** 10.2807/1560-7917.ES.2024.29.28.2400420

**Published:** 2024-07-11

**Authors:** Inna Friesen, Viola Dreyer, Angela Klingmüller, Sylvia Zuber, Ada M Hoffmann, Isabelle Suárez, Beatrice Schütz, Tim Preßel, Sönke Andres, Stefan Niemann, Jan Rybniker

**Affiliations:** 1 Research Center Borstel, Leibniz Lung Center, Supranational and National Reference Center for Mycobacteria, Borstel, Germany; 2 Research Center Borstel, Leibniz Lung Center, Molecular and Experimental Mycobacteriology, Borstel, Germany; 3 German Center for Infection Research (DZIF), Partner Site Hamburg-Lübeck-Borstel-Riems, Borstel, Germany; 4 Department I of Internal Medicine, Division of Infectious Diseases, Faculty of Medicine and University Hospital Cologne, University of Cologne, Cologne, Germany; 5 Institute for Medical Microbiology, Immunology and Hygiene, University of Cologne, Medical Faculty and University Hospital of Cologne, Cologne, Germany; 6 Klinikum Dorsten, St. Elisabeth-Krankenhaus, Dorsten, Germany; 7 Eurofins MVZ Medizinisches Labor Gelsenkirchen GmbH, Eurofins GeLaMed, Gelsenkirchen, Germany; 8 German Center for Infection Research (DZIF), Partner Site Bonn-Cologne, Cologne, Germany; 9 Center for Molecular Medicine Cologne (CMMC), University of Cologne, Cologne, Germany; * These authors contributed equally to this work and share last authorship.

**Keywords:** XDR-TB, Rifampicin, Ukraine, MDR-TB, GeneXpert, I491F

## Abstract

This report documents the case of a Ukrainian patient infected with an extensively drug-resistant (XDR) lineage 2 *Mycobacterium tuberculosis* strain harbouring the rifampicin resistance mutation RpoB I491F. This mutation is not detected by routine molecular WHO-recommended rapid diagnostics, complicating the detection and treatment of these strains. The occurrence of such mutations underscores the need for enhanced diagnostic techniques and tailored treatment regimens, especially in eastern Europe where lineage 2 strains and XDR-tuberculosis are prevalent.

Tuberculosis (TB) is a highly infectious disease caused by *Mycobacterium tuberculosis* complex (MTBC) strains that remains a significant global health issue. The World Health Organization (WHO) has estimated 10 million new TB cases and 1.5 million TB related deaths in 2022 alone [1]. The challenge of TB management is exacerbated by the emergence of drug-resistant strains, including multidrug-resistant TB (MDR-TB, resistance at least to isoniazid (INH) and rifampicin (RIF)) and extensively drug-resistant TB (XDR-TB, MDR-TB plus resistance to any fluoroquinolone (FLQ) and at least any other WHO Group A drug, such as bedaquiline (BDQ) or linezolid (LZD)). This report describes a Ukrainian patient infected with a MTBC Beijing/lineage 2 strain harbouring the RpoB I491F mutation and a mutation in *rv0678*. 

## Case description

In September 2023, a patient in her 30s travelled from Ukraine to Germany, where she lived in a refugee accommodation. In October 2023, she presented with symptoms of cough, fever, headaches and weight loss at a German hospital. The patient reported that she had been treated for pulmonary TB in Ukraine in 2015 with a standard four-drug regimen. Treatment with RIF was stopped after 2 months due to nausea, and INH, ethambutol (EMB) and pyrazinamide (PZA) were continued for a total of 6 months. Details regarding the resistance pattern of the strain isolated in 2015 are not available.

Chest computed tomography performed in 2023 revealed extensive cavitary lesions in the lungs. Acid-fast bacilli were detected in sputum and bronchoalveolar lavage fluid. The Xpert MTB/RIF Ultra (Cepheid) test was positive for *M. tuberculosis* but negative for RIF resistance, leading to the initiation of a standard four-drug anti-tuberculous therapy. Despite treatment initiation, the patient developed haemoptysis that responded to conservative treatment. However, the patient continued to experience recurrent episodes of fever and nausea.

Subsequent phenotypic resistance profiling of cultured *M. tuberculosis* revealed drug resistance above the critical concentration (CC) for INH, PZA and EMB. Resistance to RIF was at the critical concentration of 0.5 mg/L and was reported to the clinicians as susceptible. This finding rendered the initial treatment regimen ineffective. Consequently, the patient was transferred to the Division of Infectious Diseases at the University Hospital Cologne for further treatment. The isolate was shipped to the Supranational Reference Center for Mycobacteria (Research Center Borstel) for additional testing.

Targeted sequencing (Sanger sequencing) revealed a mutation in RpoB I572F, which, according to *Escherichia coli* nomenclature, translates to I491F. Consecutive whole genome sequencing identified the isolate as a lineage 2/Beijing strain and predicted resistance to multiple drugs, including INH, RMP, EMB, PZA, FLQ, BDQ, clofazimine (CFZ) and LZD (Table).

**Table:** Phenotypic drug susceptibility testing and whole genome sequencing results, first cultured (baseline) *Mycobacterium tuberculosis* isolate, Germany, October 2023

Antibiotic	Mutation	Interpretation	MIC (MGIT) mg/L
Isoniazid	*fabG1* -15c > t	R	> 1
	KatG S315T	R	
Rifampicin	RpoB I491F	R	0,5
Rifabutin	RpoB I491F	R	0,5
Ethambutol	EmbB M306I	R	> 5
Pyrazinamide	*pncA* 73_del_g	R	> 100
Moxifloxacin	GyrA D94G	R	> 1
Levofloxacin	GyrA D94G	R	> 1
Bedaquiline	*rv0678* 138_ins_g *rv0678* 193_del_g	R	2
		R	
Clofazimine	*rv0678* 138_ins_g *rv0678* 193_del_g	R	> 1
		R	
Linezolid	RplC C154R	R	1
D-cycloserine	None	S	≤ 16
Delamanid	None	S	≤ 0,06
Amikacin	None	S	≤ 1
Pretomanid	None	S	≤ 0,5

del: deletion; ins: insertion; MGIT: mycobacterial growth indicator tube; MIC: minimal inhibitory concentration; R: resistant; S: susceptible.

Antimicrobial susceptibility testing was performed with BD BACTEC MGIT960.

In addition, the Supranational Reference Center for Mycobacteria (Research Center Borstel) performed antibiotic susceptibility testing (AST) using an automated mycobacterial culture testing system (BD BACTEC MGIT960). Phenotypic susceptibility testing confirmed low-level rifampicin resistance with minimum inhibitory concentration (MIC) results for RIF and rifabutin (RFB) at the CC, and resistance above the CC for INH, EMB, PZA, FLQ, BDQ and CFZ, identifying this strain as XDR. The baseline isolate was susceptible to delamanid (DLM), pretomanid (PTM), d-cycloserine and amikacin (AMK) at their respective critical concentrations recently recommended by the WHO [2] (Table).

The patient is currently treated with a regimen including AMK, PTM, meropenem plus clavulanic acid, RFB and terizidone. Recently published data regarding the use of PTM in a non-BPaLM regimen encouraged us to use PTM rather than DLM [3]. Due to low-level resistance to rifamycins, we decided to include RFB to the regimen. The choice of RFB rather than RIF was based on data indicating that PTM clearance is less pronounced when combined with RFB [3,4]. The patient showed good clinical response, and culture conversion was achieved after 6 months of treatment.

## Epidemiology of drug resistant tuberculosis

The WHO European Region accounts for only 2% of all TB cases and has, on average, a low incidence of 25 cases per 100,000 [1]. However, the region includes countries such as Kyrgyzstan (130/100,000) and Ukraine (90/100,000) with an incidence rate that is considerably higher than the average. Globally, the WHO estimated that 410,000 people developed MDR-TB or at least RIF-resistant TB in 2022 (5.2/100,000), with high rates of MDR-TB in several eastern European countries such as Ukraine, which has an incidence (30/100,000) above the average [1].

A recent concern is the emergence of MTBC strains with a particular RpoB mutation, I491F, predominantly found in lineage 4 strains of the sub-Saharan African region. The I491F mutation is highly dominant in Eswatini and has been reported occasionally in other countries [5-10]. This mutation is problematic because it is not detected by any of the molecular WHO-recommended rapid diagnostics [6,10]. The I491F mutation leads to misdiagnosis of patients with RIF-resistant (RR), MDR- and XDR-TB as susceptible cases. Initiation of an ineffective treatment regimen increases the risk of treatment failure, further resistance amplification and transmission. Strains with the RpoB I491F mutation have a borderline RIF resistance, potentially also rendering the detection by phenotypic AST difficult [11]. The presence of the RpoB I491F mutation in strains of other lineages is particularly concerning, as it indicates that this problematic mutation is not confined to lineage 4 strains. In addition, it has been shown that RpoB I491F outbreak strains are more likely to harbour mutations in *rv0678* [9,10], a gene encoding the repressor of the *MmpS5-MmpL5* efflux pump. Mutations in *rv0678* can lead to increased efflux of BDQ [12], resulting in higher MICs and reduced drug efficacy.

## Discussion

This case highlights the need for enhanced diagnostic techniques to detect low-level rifampicin resistance, particularly in strains harbouring the RpoB I491F mutation. 

The presence of the RpoB I491F mutation in the Beijing/lineage 2 strain is a concern, as it signifies the potential spread of this mutation beyond lineage 4 strains, further complicating the TB resistance landscape. Lineage 2 strains are predominantly found in eastern Europe, a region already experiencing a large number of XDR-TB cases [13]. The prevalence of these strains in eastern Europe raises considerable public health concerns, as it indicates the potential for widespread transmission and further entrenchment of XDR-TB in this region.

## Conclusion

The impact of the RpoB I491F mutation on the detection of RR, MDR and XDR strains underscores the importance of integrating comprehensive molecular and phenotypic methods in TB diagnostics. Moreover, the coexistence of mutations in *rv0678* poses additional challenges in managing drug resistance due to its effect on BDQ efficacy. This case illustrates the necessity for tailored treatment regimens and continuous surveillance to address the evolving landscape of TB drug resistance, especially in regions with high prevalence of lineage 2 MTBC strains.
